# Determinants of failure to progress within 2 weeks of delivery: results of a multivariable analysis approach

**DOI:** 10.1016/j.xagr.2024.100415

**Published:** 2024-10-17

**Authors:** José Morales-Roselló, Blanca Novillo-Del Álamo, Alicia Martínez-Varea

**Affiliations:** 1 Department of Obstetrics and Gynecology, La Fe University and Polytechnic Hospital, Valencia, Spain (Morales-Roselló, Novillo-Del Álamo and Martínez-Varea); 2 Department of Pediatrics, Obstetrics, and Gynecology, Faculty of Medicine, University of Valencia, Valencia, Spain (Morales-Roselló); 3 Department of Medicine, CEU Cardenal Herrera University, Castellón de la Plana, Spain (Martínez-Varea); 4 Faculty of Health Sciences, Universidad Internacional de Valencia, Valencia, Spain (Martínez-Varea)

**Keywords:** cesarean section, failure to progress, induction of labor, ultrasound

## Abstract

**Objective:**

The incidence of cesarean section (CS) for failure to progress (FP) has progressively increased; thus, knowing the factors that increase this incidence has become of crucial importance. This study aimed to find the true determinants of CS for FP within 2 weeks of delivery, proposing strategies to reduce its incidence.

**Material and Methods:**

A group of 957 term and late preterm (≥34 weeks) singleton pregnancies with a complete gestational follow-up and an ultrasound examination within 2 weeks of delivery were included in a retrospective observational study. Epidemiological, sonographic, and perinatal data were recorded, and multivariable logistic regression analyses were applied to create models to predict the importance of different variables in the explanation of FP.

**Results:**

Induction of labor was by far the most important modifiable factor, followed by smoking and maternal weight, while parity was the most important nonmodifiable factor, followed by maternal age and estimated fetal weight. The difference in days from the actual due date exerted no influence.

**Conclusions:**

To reduce the incidence of CS for FP, inductions of labor should be performed only under evidence-based medicine indications and kept to a minimum. In addition, maternal overweight reduction and maternal smoking cessation should be promoted before the initiation of gestation.

## Introduction

Failure to progress (FP) is the main indication for intrapartum cesarean section (CS), representing nearly twice of the CS due to abnormal fetal monitoring.^1^ Most studies dealing with FP have evaluated which variables predict this condition at the time of admission in women undergoing induction of labor,^2^, ^3^, ^4^, ^5^, ^6^, ^7^ and in those with spontaneous onset of labor.^1^

In general, these studies have aimed to describe the possibility of FP when there was a scarce margin to reduce its incidence. Contrarily, the purpose of the current work was not only to know the true importance of the different factors affecting FP but also to evaluate whether its incidence might be to some extent diminished by acting on the factors that could be modified throughout pregnancy.

## Material and methods

This was an observational retrospective study of singleton pregnancies followed up in the maternity department of La Fe University and Polytechnic Hospital, between March 2022 and December 2023.

Inclusion criteria included singleton pregnancies of ≥34 weeks with an ultrasound examination performed within 14 days of delivery that underwent labor contractions (due to a spontaneous onset of labor with or without premature rupture of membranes, or a labor induction) to achieve a vaginal delivery. Causes of induction included most frequently premature rupture of membranes and prolonged pregnancy and less frequently maternal age, abnormal fetal growth, maternal diabetes, gestational hypertension syndrome, and cholestasis of pregnancy. Exclusion criteria included twin pregnancies, stillbirths, and any pregnancy circumstance leading to an elective CS, as well as pregnancies with a loss of follow-up.

The following data were obtained either from the patient´s notes or at the ultrasound examination: maternal age, parity, maternal prepregnancy weight, maternal height, smoking habits, gestational age (GA), at examination, fetal sex, estimated fetal weight (EFW), EFW centile, middle cerebral artery (MCA) pulsatility index (PI) multiples of median (MoM), umbilical artery (UA) PI MoM, and cerebro-placental ratio MoM. After delivery, the medical records were revised to obtain perinatal data: onset of labor (spontaneous or induced, understanding spontaneous in cases of an uneventful labor), GA at birth, interval ultrasound to delivery (maximum of 14 days), difference in days with the estimated delivery date (EDD), via of delivery (spontaneous vaginal delivery, assisted vaginal birth, and CS due to FP or intrapartum fetal compromise (IFC), birth weight (BW), BW centile, Apgar score at 5 minutes of birth, arterial cord pH, and baby destiny (maternal ward, neonatal unit, or intensive care unit).

EFW was obtained by transabdominal ultrasound, measuring the head circumference, biparietal diameter, abdominal circumference, and femur length according to Hadlock's equation.^8^ The UA and MCA were evaluated using color and pulse Doppler. The MCA Doppler was obtained by the sphenoid wing close to the Willis circle, and the UA Doppler was obtained in a free loop of the umbilical cord. All Doppler examinations were performed using ultrasound machines with 2 to 8 MHz convex probes during fetal quiescence, in the absence of fetal tachycardia, and keeping the insonation angle with the examined vessels as small as possible. MCA PI and UA PI values were converted into MoM) dividing each value by the 50th centile (median) at each GA.^9^^,^^10^

Patients were treated according to the hospital protocol, with no changes due to the study. Concerning inductions, in the case of a favorable Bishop score at admission (≥6), direct oxytocic induction (Syntocynon) was carried out, while in case of an unfavorable Bishop at admission (<6), an initial cervical ripening with PgE2, dinoprostone (Propess), or a mechanical balloon (Cook) was used up to 12 hours. The mechanical balloon was applied in cases of previous CS, fetal growth restriction, smallness for GA, and maternal asthma or cardiopathy. Finally, if cervical ripening was unsuccessful, it was followed by oxytocic induction.^11^

CS for IFC was defined as a CS indicated for an abnormal cardiotocogram or a fetal scalp pH below 7.2, while CS for FP included three scenarios, in line with the national and hospital guidelines^11^:

1. Failure of induction defined as the failure to reach the active period of labor (4 cm of dilatation and complete cervical effacement) after 12 hours of oxytocic induction and regular uterine contractions (at least four every 10 minutes).
2. Arrested labor defined as the absence of progress in cervical dilatation (≥4 hours) once labor reaches the active period.
3. Cephalo-pelvic disproportion, defined as a ≥2 to 3 hours interruption of descent in multiparous and nulliparous women once a full cervical dilation has been achieved. Both limits could be amplifyed by 1 hour in women with epidural analgesia, which represents nearly the totality of our cases (from ≥2–3 to ≥3–4 hours). However, it must be indicated that to increase the possibility of descent, we used to extend the limit of 4 hours to all parturients.

Protracted labor (abnormally slow cervical dilation or fetal descent during the first or second stage of labor) was not taken into account provided delivery was finally achieved.

Descriptive statistics were performed to evaluate the population studied. Continuous variables were represented with the median and interquartile range, while categorical variables were represented with the number (*N*) and the frequency. Comparisons were made using Mann–Whitney *U* tests for continuous data and Fisher's exact tests for frequency data.

Afterward, an initial approach was done using univariable logistic regression analyses, followed by multivariable logistic regression analyses to create prediction or explanation models. The best prediction model was selected, relying on the highest area under the curve (AUC) (with the 95% confidence interval [CI], *P* value, and detection rates [DR] for a false positive rate [FPR] of 5% and 10%), but also on the lowest Akaike information criteria (AIC) which indicates the model´s simplicity and reproducibility. ROC is a probability curve and the AUC represents the degree or measure of separability. It tells how much the model is capable of distinguishing between classes. The higher the AUC, the better the model is at distinguishing between normal and abnormal cases. An excellent model has an AUC close to 1 which means it has a good measure of separability. A poor model has an AUC close to 0 which means it cannot separate normal from abnormal cases. The ROC curve is plotted with the true positive rate (DR or sensitivity) in the *Y* axis, and the FPR (1-especificity) on the *x*-axis. Despite the importance of discerning between normality and abnormality, a model must also be reproducible. To evaluate this characteristic, we used the AIC. There is generally a tradeoff between goodness of fit and parsimony: low parsimony models (ie, models with many parameters) tend to have a better fit than high parsimony models. However, adding more parameters usually results in a good model fit for the data at hand, but that same model will likely be less useful in other populations. The AIC allows a good balance between parsimony and goodness of fit. The AIC was used to select the best prediction model by means of a lower AIC, which indicated the presence of a higher accuracy. A difference in the AIC of 2 units indicated significant differences, while a difference of 2 to 4 units indicated highly significant differences.

Statistics and graphs were performed using Graph Pad Prism 9 and Stat Plus Pro 7 Free version for Apple Macintosh. Statistical significance was set at *P*<.05.

## Results

The descriptive analysis of the study population is shown in Table 1. A total of 957 patients fulfilled the inclusion criteria. In summary, the mean maternal age was 32.5 years, the mean parity was 0.62, and the mean prepregnancy weight and height were 62.5 kg and 163 cm, respectively, while half of the women were nulliparous and only 13% smoked. Considering examination, half of the fetuses were males, with a mean GA of 39.1 weeks, the mean interval examination-delivery was 6.1 days, the mean EFW and EFW centile were 3165 g, and 45.6, respectively, and the mean MCA PI MoM and UA PI MoM were close to 1. Concerning labor, half of cases were induced, at a mean GA of 40 weeks, with most babies delivered spontaneously (58%). Finally, the majority of neonates had a normal Apgar score and arterial cord pH, and only 4% of neonates needed special pediatric care.

**Table 1:** Descriptive statistics (*N* = 957)

Continuous parameters	Mean (SD)	Interquartile range
Maternal age (y)	32.5 (5.2)	33 (29, 36)
Parity	0.62 (0.82)	0 (0, 1)
Maternal prepregnancy weight (kg)	62.5 (11.8)	60 (55, 68)
Maternal height (cm)	163 (6.2)	163 (159, 167)
Maternal body mass index (kg/m^2^)	23.5 (4.2)	22,7 (20.7, 25.4)
Gestation at ultrasound (wk)	39.1 (1.1)	39.6 (38.4, 40)
EFW hadlock-4 (g)	3165 (520)	3219 (2876, 3529)
EFW hadlock-4 centile	45.6 (32.2)	44 (14, 75)
MCA PI MoM	0.97 (0.23)	0.96 (0.82, 1.12)
UA PI MoM	1.09 (0.24)	1.06 (0.92, 1.20)
Gestation at birth (wk)	40 (1.1)	40.29 (39.43, 40.86)
Difference with EDD (d)	0 (7.7)	2 (–4, 6)
Interval ultrasound-labor (d)	6.1 (3.7)	6 (3, 9)
Birth weight (g)	3242 (503)	3270 (2950, 3600)
BW centile^a^	40 (31.1)	35 (11,67)
Apgar at 5 min	9.8 (0.53)	10 (10, 10)
Arterial cord pH	7.26 (0.07)	7.27 (7.22, 7.31)

Categorical parameters	Number and frequency
Smoking	125 (13)
Gender male	479 (50)
Nulliparous	504 (52.7)
Apgar 5 min <7	10 (1.04)
Arterial cord pH <7.10	18 (1.9)
Onset of labor	
Spontaneous onset of labor	466 (48.7)
Induction of labor	491 (51.3)
Mode of birth	
Spontaneous vaginal birth	558 (58.3)
Assisted vaginal birth	222 (23.2)
Cesarean section abnormal CTG (IFC)	65 (6.8)
Cesarean section failure to progress	112 (11.7)
Neonatal special or intensive care unit	37 (3.9)

*BW*, birth weight; *CPR*, cerebroplacental ratio; *EDD*, estimated delivery date; *GA*, gestational age; *EFW*, estimated fetal weight; *FGR*, fetal growth restriction; *MoM*, multiples of the median; *SD*, standard deviation; *CTG*, cardiotocography.

a Centiles according to local population references (Hospital Clinic de Barcelona, Spain population references).*

**Figure 1:**
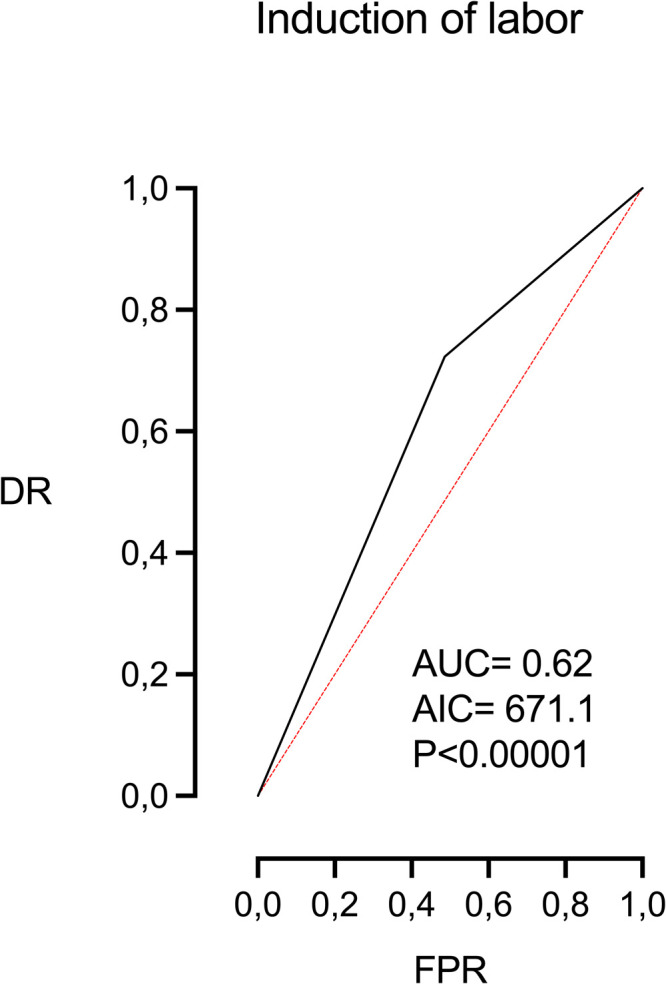
Univariable logistic regression analysis. ROC curve of induction of labor

**Figure 2:**
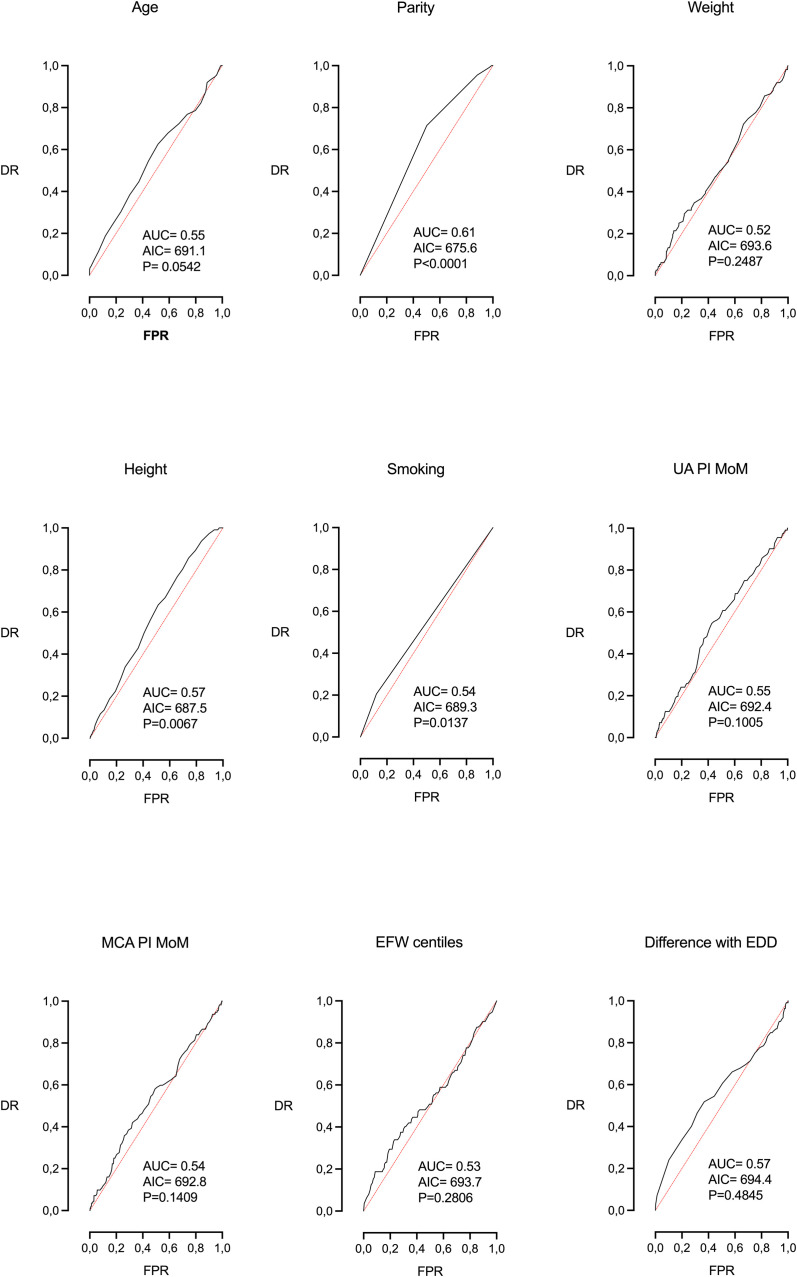
Univariable logistic regression analysis. ROC curve of the remaining parameters

Table 2 shows the univariable logistic regression analysis for the explanation of FP within 14 days of delivery. The following variables were significant or borderline significant (BS): maternal age (*P*=BS), parity (*P*<.0001), height (*P*<.01), smoking (<0.05), and induction of labor (*P*<.0001). The highest association was observed with the latter parameter (AUC=0.62, AIC=671.1) (Figure 1), followed by parity (Figure 2).

**Table 2:** Univariable logistic regression analysis for the prediction of failure to progress with 14 days of delivery

Parameter	OR	OR 5%	OR 95%	AUC	AIC	*P* value
Maternal age	1.040	0.999	1.083	0.55	691.1	BS
Parity	0.502	0.349	0.696	0.61	675.6	<.0001
Weight	1.009	0.993	1.025	0.52	693.6	NS
Height	0.957	0.926	0.988	0.57	687.5	<.01
Smoking	1.882	1.118	3.069	0.54	689.3	<.05
UA PI MoM	1.871	0.854	3.954	0.55	692.4	NS
MCA PI MoM	1.842	0.798	4.152	0.54	692.8	NS
EFW centile	1.003	0.998	1.010	0.53	693.7	NS
Difference with EDD (d)	1.010	0.984	1.038	0.57	694.4	NS
Induction of labor (yes)	2.766	1.808	4.332	0.62	671.1	<.0001

*AIC*, Akaike information criteria; *AUC*, area under the curve; *BS*, borderline significance (0.0542); *DR*, detection rate (sensitivity); *FPR*, false positive rate (1-especificity); *NS*, no statistical significance; *OR*, odds ratio.

Morales-Roselló. Determinants of failure to progress within 2 weeks of delivery. AJOG Glob Rep 2024.

Morales-Roselló. Determinants of failure to progress within 2 weeks of delivery. AJOG Glob Rep 2024.

**Figure 3:**
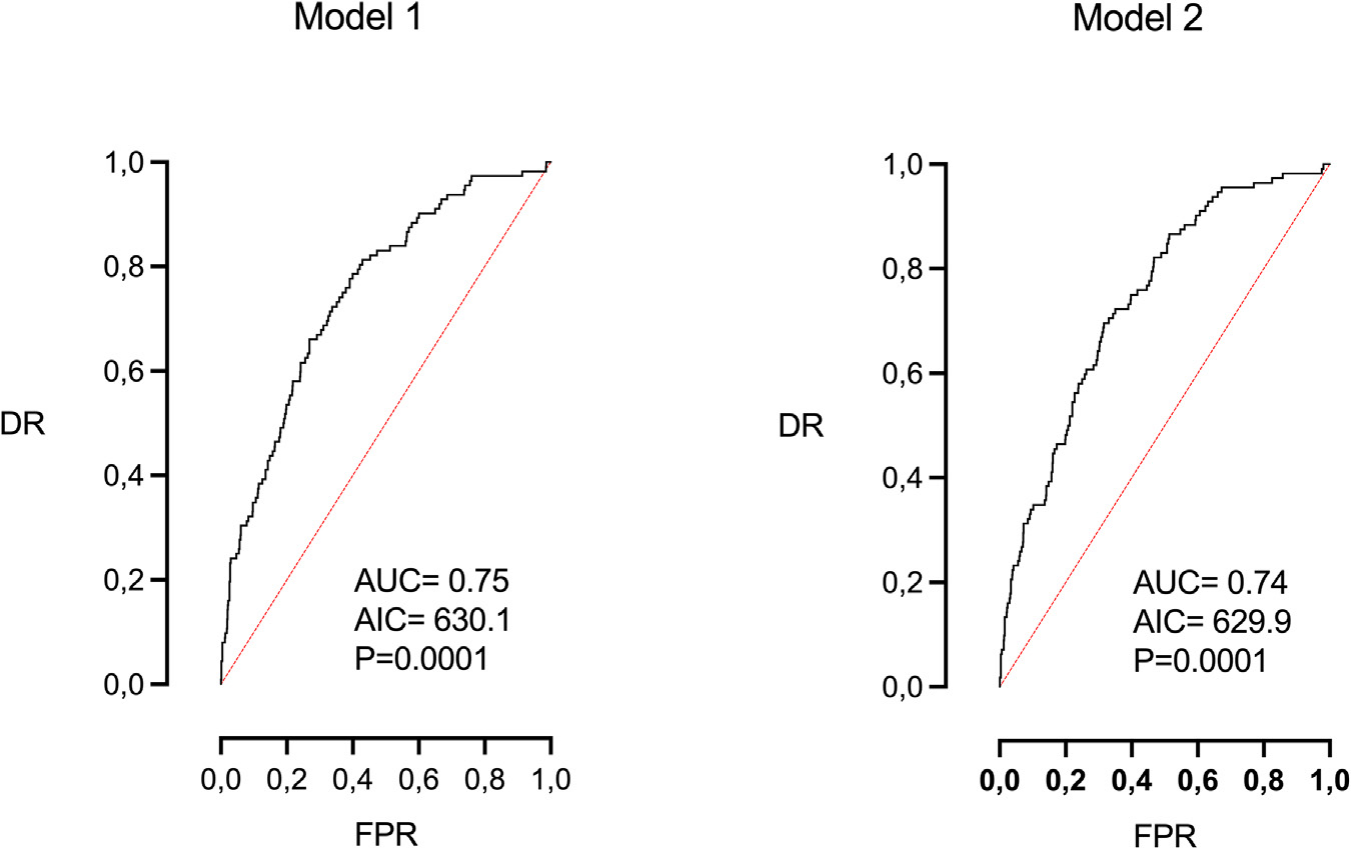
Multivariable logistic regression analysis. ROC curve of model 1 and model 2

Table 3 and Figure 3 show the multivariable logistic regression analysis. Two models are depicted. The first one includes all the study parameters (AUC=0.75, AIC=630.1, DR=25% for an FPR of 5% and 35% for an FPR of 10% (*P*<.0001), while the second includes only the parameters that were significant in the first model: maternal age, parity, maternal weight, and height, smoking habits, EFW centile, and induction of labor. This model presented a lower (better) AIC with a similar AUC using fewer parameters. However, the prediction ability was again moderate (AUC=0.74, AIC=626.9, DR=30% for an FPR of 5%, and 33% for an FPR of 10%, *P*<.0001). In this model, similarly to model 1, the most important parameters were induction of labor (OR 2.8, *P*<.0001), and parity (OR 0.45, *P*<.0001). Neither Doppler nor the difference in days from the EDD proved to be significant.

**Table 3:** Multivariable logistic regression analysis for the prediction of failure to progress with 14 days of delivery

Parameter	Estimate	Standard error	OR	OR 5%	OR 95%	*P* value
Model 1 (all parameters)						
Maternal age	0.059	0.022	1.061	1.017	1.107	<.01
Parity	–0.819	0.183	0.440	0.308	0.631	<.00001
Weight	0.021	0.008	1.021	1.004	1.039	<.05
Height	–0.080	0.018	0.923	0.889	0.957	<.0001
Smoking	0.770	0.278	2.161	1.253	3.727	<.01
UA Pi MoM	0.710	0.442	2.034	0.854	4.843	NS
MCA PI MoM	0.825	0.466	2.283	0.915	5.692	NS
EFW centile	0.010	0.003	1.011	1.003	1.018	<.01
Difference with EDD (d)	0.002	0.014	1.002	0.974	1.030	NS
Induction of labor (yes)	1.028	0.237	2.796	1.755	4.455	<.0001
Intercept	5.230					
AUC=0.75, (95% CI=0.70, 0.80), AIC=630.1, DR=25% for an FPR of 5% and 35% for an FPR of 10%, *P*<.0001.						
Model 2 (significant parameters in model 1)						
Maternal age	0.055	0.021	1.056	1.012	1.102	<.05
Parity	–0.786	0.180	0.455	0.320	0.648	<.0001
Weight	0.021	0.008	1.021	1.004	1.039	<.05
Height	–0.078	0.018	0.924	0.891	0.958	<.0001
Smoking	0.784	0.275	2.192	1.276	3.764	<.01
EFW centile	0.010	0.003	1.010	1.003	1.017	<.01
Induction of labor (yes)	1.033	0.236	2.809	1.766	4.468	<.0001
Intercept	6.664					
AUC=0.74, (95% CI=0.69, 0.79), AIC=629.9, DR=30% for an FPR of 5% and 33% for an FPR of 10%, *P*<.0001.						

*AIC*, Akaike information criteria; *AUC*, area under the curve; *DR*, detection rate (sensitivity); *FPR*, false positive rate (1-especificity); *OR*, odds ratio.

Morales-Roselló. Determinants of failure to progress within 2 weeks of delivery. AJOG Glob Rep 2024.

## Discussion

This study shows, using multivariable analysis, the importance of different factors in the explanation of FP.

From a management perspective, these factors were divided into modifiable factors (those that could be changed at any stage, either performing/avoiding them or increasing/reducing their intensity, and not-modifiable factors (those that could not be modified at any stage).

***A. Modifiable factors***.

The main modifiable factor by far was the induction of labor, with an OR (95% CI) of 2.81 (1.77, 4.47) *P*<.0001 in both models. This OR proved that regardless of any other consideration, the practice of induction nearly tripled the possibility of FP. A practical consequence of this finding would be that induction of labor should only be considered when the benefit of a prompt vaginal delivery clearly surpasses the maternal and/or fetal risks of waiting for a spontaneous onset.^12^ This rationale of waiting for a spontaneous delivery is supported by the fact that the difference in days from the EDD did not exert any influence on the occurrence of FP, and therefore, no adverse consequences might be expected in waiting for the spontaneous onset of labor. This is in line with the common practice of not inducing pregnancies before 41 weeks, since there is insufficient evidence to expect any benefit from an early induction in the general population.^13^, ^14^, ^15^ In this regard, although some trials have advocated for a general induction at 39 weeks (ARRIVE),^16^, ^17^, ^18^ results are still controversial, and some studies still associate the widespread practice of induction with an increase in the CS incidence.^19^ Our data are in line with this rationale. Moreover, we consider that there is a margin to reduce the practice of induction in many other circumstances that have not clearly proven to place the fetus at a higher risk of adverse perinatal outcome, like the induction in healthy older mothers (≥40 years) with normal cardiotocography, and normal Doppler ultrasound,^1^^,^^20^ or the induction in fetuses with macrosomia in whom induction of labor has not proved to cause any benefit.^21^^,^^22^ We could also include other conditions in this list, like premature rupture of membranes, fetal cholestasis, gastroschisis, etc. In fact, evidence only supports induction in post-term pregnancy, although the evidence is weak regarding the timing, (41 vs 42 weeks), and in women with hypertensive disorders of pregnancy. Evidence is unfortunately weak in women with term rupture of membranes. For all other indications, like fetal gastroschisis, there is also insufficient power to draw clear conclusions.^23^^,^^24^

The second modifiable parameter in our list was smoking. Patients with smoking habits showed more than twice the rate of FP with an OR (95% CI) of 2.19 (1.28, 3.76) (*P*<.01). It is known that smoking during pregnancy leads to a reduction in the EFW and BW.^25^ This might paradoxically be a reason for an easier delivery and a decrease in the frequency of FP. Contrarily, our results show a higher risk of FP for CS. However, as animal experimentation suggests, this might be caused by a different mechanism, like a less efficient cervical ripening.^26^ Whatever the mechanism, considering its independent influence on FP, women should try to get pregnant after quitting smoking.

The third modifiable parameter on our list, although of much lesser importance, was the maternal weight prior to pregnancy (OR 1.02). Although it may be argued that this is a prepregnancy objective, we included it in the group considering that weight reduction prior to labor was a plausible objective with an evident influence on labor. This finding was in line with earlier studies, which found a relationship between prepregnancy overweight and intrapartum CS.^27^, ^28^, ^29^

Therefore, considering its independent (although small) influence on FP, women should try to start conception by avoiding being overweight.

***B. Nonmodifiable factors***.

The most notorious nonmodifiable factor was parity, drastically halving the FP rate with an OR (95% CI) of 0.44 (0.32, 0.65) (*P*<.0001). This meant that, in general, women with a previous sibling presented half of the possibilities to present CS for FP. This is in line with many previous works that have underlined the association of parity with CS for FP, failure of vacuum, assisted vaginal delivery, and shoulder dystocia.^7^^,^^30^^,^^31^

The second nonmodifiable factor was maternal height, with an OR (95% CI) of 0.92 (0.89, 0.96) (*P*<.0001). This meant that, in comparison with taller women, shorter women could expect a 10% increase in the possibility of CS. This is in line with previous evidence of an association between maternal height and CS for FP.^7^^,^^32^^,^^33^

Finally, other less important nonmodificable variables were maternal age and EFW. Maternal age, with an OR (95% CI) of 1.06 (1.01, 1.10) (*P*<.05), was a minor parameter. According to the OR, older women should expect a 5% increase in their risk, much lower than parity. However, it should be taken into account when planning induction in otherwise older women with a normal cardiotocogram and Doppler ultrasound examination, as the risk of induction would be slightly increased by the risk of maternal age. This is in line with previous studies, which indicate an independent association between maternal age and CS for FP.^34^^,^^35^ EFW, with an OR (95% CI) of 1.01 (1, 1.2) (*P*<.01), was also a minor parameter. According to its OR, the risk of FP directly caused by the EFW centile would be extremely small. At first glance, this would seem controversial, as the bigger a fetus is, the more difficult it is to deliver. However, most obstetricians would accept that tall women with previous deliveries are usually able to deliver large babies, provided the labor initiates spontaneously, so it might be argued that FP in babies with a high EFW centile might not be entirely attributable to the EFW centile itself but to the presence of accompanying determinants like nulliparity, induction of labor, or short stature. Although EFW is largely influenced by maternal glucose levels, we did not consider it a modifiable factor as maternal postprandial glucose control cannot fully correct it. Another consequence of this finding was drawn from the multivariable analysis. As it shows, the EFW centile is much less important than the induction of labor (OR 2.81 vs 1.01). Thus, this data would not support induction of labor to avoid changes in the EFW, as the increase in EFW over a few days would not be as marked as to outweigh the stronger risk increase produced by induction, with the consequent benefit of being able to trigger a spontaneous onset of labor during those days. In addition, there is a high likelihood (>50%) of overestimating the EFW in suspected macrosomia.^36^^,^^37^ Moreover, the most recent update of the NICE guidelines suggests that the risk of perinatal death, brachial plexus injuries in the baby, or the need for emergency CS was not different between the induction and the expectant management.^38^ Therefore, considering the impact of induction on the birth experience, they advice informing the patient and discussing all options for a consensuous decision,^38^ Our results support this and other guidelines that do not recommend induction for suspected macrosomia.^13^, ^14^, ^15^^,^^39^

Other studied variables with no influence on FP were the MCA and UA PI Doppler. This is in agreement with earlier publications, in which Doppler was related to CS for IFC and not to CS for FP.^40^

Considering all this information, the authors advocate for the strategy of avoiding labor inductions to reduce FP in controlled gestations with normal follow-up, weighing the risks and benefits of indications whose benefits have not been proven. We acknowledge some limitations in the study, such as the need for internal and external validation to extrapolate the data; and the different definitions of FP in the other guidelines.

In conclusion, induction of labor is the modifiable factor with the greatest influence on the rate of FP; followed by smoking, and maternal prepregnancy weight. Parity and maternal age are the most important nonmodifiable factors. A policy to reduce inductions, avoid maternal overweight prior to pregnancy, and cessation of smoking should be recommended to decrease the rate of CS due to FP.

## CRediT authorship contribution statement

**José Morales-Roselló:** Writing – review & editing, Validation, Supervision, Resources, Methodology, Investigation, Funding acquisition, Formal analysis, Data curation, Conceptualization. **Blanca Novillo-Del Álamo:** Writing – review & editing, Writing – original draft, Investigation, Conceptualization. **Alicia Martínez-Varea:** Writing – review & editing.
